# Species-Specific Variation in Abscisic Acid Homeostasis and Responses Impacts Important Traits in *Crassocephalum* Orphan Crops

**DOI:** 10.3389/fpls.2022.923421

**Published:** 2022-07-12

**Authors:** Adebimpe N. Adedeji-Badmus, Sebastian Schramm, Michael Gigl, Williams Iwebema, Pablo Albertos, Corinna Dawid, Tobias Sieberer, Brigitte Poppenberger

**Affiliations:** ^1^Biotechnology of Horticultural Crops, TUM School of Life Sciences, Technical University of Munich, Freising, Germany; ^2^Chair of Food Chemistry and Molecular Sensory Science, TUM School of Life Sciences, Technical University of Munich, Freising, Germany; ^3^Research Group Plant Growth Regulation, TUM School of Life Sciences, Technical University of Munich, Freising, Germany

**Keywords:** ABA, dormancy, drought stress, ebolo, neglected crop, redflower ragleaf

## Abstract

*Crassocephalum rubens* and *Crassocephalum crepidioides* are plant species native to Africa, but grow in most tropical and subtropical regions of the world. They are rich in vitamins, minerals, and essential oils and are traditional leafy vegetables and medicinal plants in Sub-Saharan Africa. The plants are still mainly collected from the wild but shall be taken into cultivation and an important aim in the domestication of these species is to improve traits that are relevant for crop production. Here, seed formation and germination capacities in *C. crepidioides* and *C. rubens* were investigated, and it was found that *C. crepidioides* exhibits a higher level of seed dormancy, which could be broken with light, and was correlated with higher amounts of abscisic acid (ABA), a plant hormone that promotes seed dormancy. ABA is also very well-known for its role in abiotic stress tolerance, and it is shown that tetraploid *C. crepidioides* exhibits a higher level of resistance against drought and heat stress than diploid *C. rubens*, traits that will benefit the cultivation of these plants, particularly in rain-fed cropping systems. The potential of *Crassocephalum* to improve nutrition and increase the resilience of marginal cropping systems in Africa is discussed.

## Introduction

More than 50,000 edible plants exist on our planet, but only a small fraction of them have been domesticated and taken under cultivation (Gruber, 2017). In addition to the major and many minor crops, wild and semi-wild species can be of high regional importance as food sources. Such wild, edible plants, with crop-like traits, often have a long tradition of use, contribute to nutrition and food security, and have roles in natural medicine. However, when demands are high, over-exploitation can threaten the natural populations (Corlett, 2016) and thus the cultivation of such plants could protect ecosystems. Moreover, stronger use of the wild and orphan crops is seen as a strategy to improve the resilience of marginal cropping systems as a climate change adaptation strategy (Renard and Tilman, 2019); since they are usually natives, that are well-adapted to the local environmental conditions.

Orphan crops have low economic importance on a global scale and thus research on them has been neglected by industry, the international scientific community, and funders, resulting in a poor understanding of their cultivation and a lack of genetic improvement. To improve this situation, the African Orphan Crops Consortium was founded, an initiative that aims to sequence the genomes of 101 orphan crops of Africa with a high potential to improve food security on the continent and beyond (Hendre et al., 2019; Jamnadass et al., 2020). One of the selected species, is *Crassocephalum rubens*, an annual plant that belongs to the Senecioninae, a tribus of the Asteraceae subfamily Asteroidae (Pelser et al., 2007). It is native to Sub-Saharan Africa and together with its close relative *Crassocephalum crepidioides* is used as a leafy vegetable and medicinal plant in African countries such as Nigeria and Benin (Adjatin et al., 2013; Oyebode et al., 2019). English names are ebolo, redflower ragleaf, or fireweed, the latter name reflecting the fact that it is a weed in tropical and subtropical regions of the world (Chen et al., 2009).

These plants are interesting to humans for the following two main reasons: (1) Their high potential as nutritious crops that perform well even in marginal conditions (Hung et al., 2019; Thakur et al., 2019; Wijaya et al., 2011) and (2) their medicinal properties, which include an ability of plant extracts to suppress the growth of cancer cells in cell culture (Dairo and Adanlawo, 2007; Hou et al., 2007; Tomimori et al., 2012; Oyebode et al., 2019). In addition, *C. crepidioides* is studied to understand its invasive nature and properties as a weed (Chen et al., 2009; Yuan and Wen, 2018).

While *C. rubens* is diploid, *C. crepidioides* is tetraploid and has a genome size of 12.3 Gbp (Schramm et al., 2021). Polyploidy is a major driving force of evolution (Soltis and Soltis, 2009; Chen, 2010) and polyploids often have competitive advantages over their diploid relatives, which include increased plant vigor, higher yields, and a richer diversity of secondary metabolites that polyploids can form (Comai, 2005; te Beest et al., 2011; Mayfield et al., 2011; Wei et al., 2019). In this regard, it was recently shown that in specific growth conditions, such as under nitrogen starvation, *C. crepidioides* can synthesize high amounts of the phyrrolizidine alkaloid (PA) jacobine, an ability that the diploid *C. rubens* appears to lack (Rozhon et al., 2018; Schramm et al., 2021). Phyrrolizidine alkaloid are chemical defense compounds produced to restrict herbivore feeding and are toxic to humans. Thus, as an important step in the domestication of *C. crepidioides* PAs, as an anti-nutritional trait, require removal (Schramm et al., 2021).

Another trait that requires attention is the seed viability and germination capacities, which can limit the cultivation of *C*. *crepidioides* (Sakpere et al., 2013). The plant is a pioneer species that forms plenty of seeds dispersed by wind, rapidly colonizes open areas, and there was the first evidence that *C. crepidioides* has a light requirement for germination (Sakpere et al., 2013; Yuan and Wen, 2018). Here, the seed formation and germination capacities in *C. crepidioides* and *C. rubens* were investigated, and it was found that *C. crepidioides* exhibits a higher level of seed dormancy, which was correlated with higher amounts of abscisic acid (ABA), a plant hormone that promotes seed dormancy, in dry seeds. ABA is also very well-known for its ability to improve tolerance against certain abiotic stress types, and it is shown that *C. crepidioides* exhibits a higher level of resistance against drought and heat stress than *C. rubens*, traits that are beneficial for the cultivation of these plants, in particular in rain-fed cropping systems.

## Materials and Methods

### Plant Material and Growth Conditions in the Soil

All ecotypes of *C. crepidioides* and *C. rubens* used in this study, namely, *C.c*.Ile-Ife, *C.c*.Osogbo, *C.c*.Nepal, *C.c*.Thailand, *C.r.*Mali, and *C.r*.Burkina Faso, were described before (Rozhon et al., 2018; Schramm et al., 2021). For cultivation in soil, soil substrate C700 with Cocopor^®^ (Stender AG, Schermbeck, Germany) was used and the plants were grown in chambers (Bright Boy, CLF Plant Climatics, Wertingen), equipped with Philips Master TL-D 58W/840 light bulbs. The standard growth conditions were a temperature of 25 ± 2°C and cycles of 8 h of darkness and 16 h of white light, with an intensity of 80 μmol m^–2^ s^–1^. For growth in the greenhouse, the seeds were germinated in the growth chambers, using the conditions described at above, and transferred to the greenhouse for 3–4 weeks, where they were grown at a temperature of 25 ± 3°C in the summer or 20 ± 2°C, and with additional artificial light (80–100 μmol/m^–2^ s^–1^, for 12 h daily) in the winter, and with a relative humidity of 50%.

### Seed Treatments and Germination Experiments

For seed analyses, seeds from mother plants grown in the greenhouse were collected at the point of dispersal. They were cleaned, air-dried at room temperature, and stored in paper bags. For germination experiments, seeds from mother plants grown at the same time were sterilized for 20 min in a 75% commercial bleach solution containing 0.01% Triton X-100 and then rinsed 3 times with deionized water. They were plated on half-strength Murashige and Skoog (1/2 MS) medium (Duchefa, Haarlem, Netherland) and incubated in growth chambers at 21 or 25°C in the dark or in long days of 16 h of white light at the required light intensities. The seed germination was assessed after 14 days of incubation and was considered completed when the radical had emerged from the seed coat.

To test the effect of hormones, hormone biosynthesis inhibitors or osmolytes on germination capacities, GA_3_ (Duchefa, Haarlem, Netherlands), 24-epiBrassinolide (Apollo Scientific, Cheshire, United Kingdom), (+)-*cis*,*trans*-ABA (Duchefa, Haarlem, Netherlands), Fluridone (Sigma, St Louis, MO, United States), brassinazole, and NaCl or D-Mannitol (Sigma–Aldrich, Steinheim, Germany) were dissolved in dimethyl sulfoxide and added at different concentrations to the 1/2 MS medium.

### Drought Tolerance and Detached Leaf Water Loss Assays

The drought tolerance assays were performed with adult plants with comparable numbers of leaves, using 7-week-old plants of *C. rubens* Mali and 8-week-old plants of *C. crepidioides* Ile-Ife, which were grown in the incubators in standard growth conditions (25 ± 2°C and long days of 8-h dark/16 h white light, with an intensity of 80 μmol m^–2^ s^–1^). The plants were set to comparable watering levels at day 0 and then subjected to progressive drought by withholding water for 7 days before they were re-watered. The survival rates were determined after 3 days of recovery and were defined as the ability to form new leaves.

For detached leaf water loss assays, the third pair of true leaves of 4-week-old plants of *C. rubens* Mali and 5-week-old plants of *C. crepidioides* Ile-Ife was detached, placed on an open petri dish, and incubated in room temperature. The leaves were weighed at 1 h intervals for 5 h to determine the rate of water loss.

### Heat Stress and Frost Tolerance Experiments

*Crassocephalum crepidioides* Ile-Ife and *C. rubens* Mali were grown for 4 weeks in incubators set to standard growth conditions of 25 ± 2°C and long days (8-h dark/16 h white light, with an intensity of 80 μmol m^–2^ s^–1^). They were then directly exposed to a temperature of 45°C using an incubator (MIR-154, Panasonic, Japan) and a duration of 2–8 h, before returning to 25°C. Survival was analyzed after 2 weeks of recovery and was defined as the ability to produce new leaves from an intact shoot apical meristem (SAM).

Frost tolerance assays were also performed with 4-week-old plants pre-grown in the Bright Boy incubators at 25 ± 2°C on long days, but by moving them directly to chilling and freezing temperatures of + 4°C to –4°C for 3 h in an incubator (MIR-154, Panasonic, Japan), before returning them to 25°C. Survival was analyzed after 2 weeks of recovery and was defined as the ability to produce new leaves from an intact SAM.

### Quantification of Abscisic Acid by Liquid Chromatography Tandem Mass Spectrometry

The following chemicals were obtained commercially: ABA and ABA-d_6_ (Sigma–Aldrich, Steinheim, Germany); phaseic acid and dihydrophaseic acid (National Research Council Canada, Ottawa, Canada); and ABA glucose ester (Olchemim, Olomouc, Czech Republic). Formic acid was purchased from Merck (Darmstadt, Germany); ethyl acetate and acetonitrile (LC-MS grade) were obtained from Honeywell (Seelze, Germany). Water used for chromatography was prepared using a MiliQ Reference A + Water Purification System (Milipore, Schwalbach, Germany).

Abscisic acid, phaseic acid (PA), dihydrophaseic acid (DPA), and ABA glucose ester (ABA-GE) were quantified from dry seeds, imbibed seeds, and young seedling, and from drought-exposed plants according to Chaudhary et al. (2020) and Abramov et al. (2021). Dry seeds came from seed batches grown in the greenhouse in summer and were grounded directly. To generate material from imbibed seeds, the seeds were imbibed on filter paper for 2 days at 25°C and in the dark. The samples of young seedlings came from 2 to 3-day-old plants with fully expanded cotyledons, which were germinated on filter paper at a temperature of 25°C and grown under long-day conditions (16 h of light/8 h of darkness).

All samples were homogenized in liquid nitrogen and 150–200 mg of material were transferred to a bead beater tube (2 ml, CKMix, Bertin Technologies, Montigny-le-Bretonneux, France), filled with ceramic balls (zirconium oxide; mixed beads with 1.4- and 2.8-mm diameter). Then, ethyl acetate (1 ml) was added, and the samples were spiked with an internal standard solution (20 μl) containing (+)-*cis*,*trans*-abscisic acid d_6_ (2.5 μg/ml) acetonitrile. Phytohormone extraction was performed by grinding (3 × 30 s with 30 s breaks; 6,000 rpm) using a bead beater homogenizer (Precellys Homogenizer, Bertin Technologies, Montigny-le-Bretonneux, France). The resulting supernatants were membrane filtered, evaporated to dryness, and resumed in acetonitrile (70 μl). Aliquots (2 μl) of all samples were injected into the ultra high performance liquid chromatography (UHPLC)-MS/MS system.

A QTrap 6500 + mass spectrometer (Sciex, Darmstadt, Germany) was used to obtain electrospray ionization (ESI) mass spectra and product ion spectra. The MS/MS system was operated in the scheduled multiple reaction monitoring (MRM) and ESI negative mode. The ion source parameters determined were as follows: ion spray voltage (–4,500 V), curtain gas (35 psi), temperature (550°C), gas 1 (55 psi), gas 2 (65 psi), collision-activated dissociation (-3 V), and entrance potential (–10 V). For the hormone analysis, first, the MS/MS parameters were tuned to achieve fragmentation of the [M-H]^–^ molecular ions into specific product ions as listed in the following: (+)*cis*,*trans* abscisic acid 263→153 (quantifier) and 263→219 (qualifier); (+)-*cis*,*trans*-abscisic acid d_6_ 269→159 (quantifier) and 269→225 (qualifier); dihydrophaseic acid 281→237 (quantifier) and 281→171 (qualifier); phaseic acid 279→139 (quantifier) and 279→205 (qualifier); ABA glucose ester 425→263 (quantifier) and 425→153 (qualifier).

The mass spectrometer was connected to an ExionLC ultra high performance liquid chromatography (UHPLC) system (Shimadzu, Duisburg, Germany) consisting of two ExionLC AD pumps, an ExionLC degasser, an ExionLC AD autosampler, an ExionLC AC column oven, and an ExionLC controller. Chromatographic separation was achieved with a Kinetex F5 column (100 × 2.1 mm, 100 Å, 1.7 μm, Phenomenex, Aschaffenburg, Germany) operated with a flow rate of 0.4 ml/min and a column oven temperature of 40°C. The solvents consisted of 0.1% formic acid in water (v/v) as solvent A and 0.1% formic acid in acetonitrile (v/v) as solvent B. Chromatography was performed with the following gradient: 0% B held for 2 min, increased in 1 min to 30% B, in 12 min to 30% B, increased in 0.5 min to 100% B, held 2 min isocratically at 100% B, decreased in 0.5 min to 0% B, and held 3 min at 0% B. Instrument control and data acquisition were performed with Analyst 1.6.3 software (Sciex, Darmstadt, Germany). Phytohormone quantification was performed based on comparison with standard curves prepared with reference compounds and expressed as nanogram of hormone per gram of fresh weight of the samples.

### Scanning Electron Microscopy

For scanning electron microscopic images of buds and flowers, the tissues were shock-frozen in liquid nitrogen, placed on a metal support rack, and visualized with a Hitachi TM3000 scanning electron microscope (Hitachi High-Tech, Tokyo, Japan).

## Results

### *Crassocephalum crepidioides* and *Crassocephalum rubens* Show Differences in Flower Morphology and Seed Development

**FIGURE 1:**
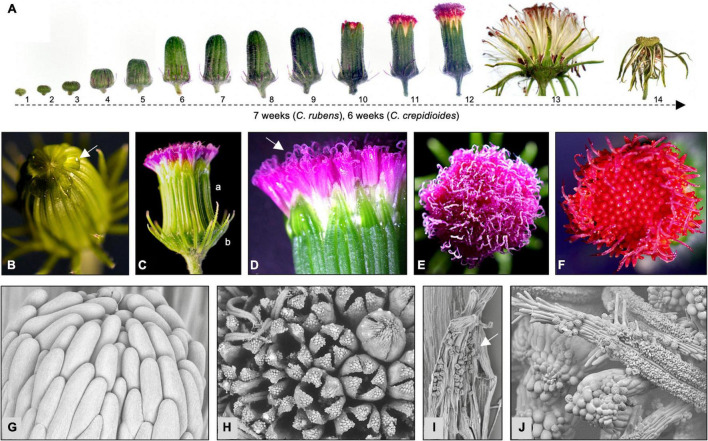
*Crassocephalum rubens* and *C. crepidioides* form composite flowers, whose disk florets differ in corolla color. **(A)** Stages of flower development of *C. crepidioides* Ile-Ife. **(B–E)** Flower details of *C. rubens* Mali and *C. crepidioides* Ile-Ife **(F)**. Nectarous secretions on the tip of a capitulum prior to anthesis at stage 9 **(B)**. Side view of a capitulum after anthesis showing the outer (a) and inner (b) involucral bracts **(C)**. Side view of a capitulum with open florets and emerged stigmas **(D)**. Top view of a capitulum of *C. rubens* at stage 12 **(E)**. **(F)** Top view of a capitulum of *C. crepidioides* at stage 11. **(G–I)** Scanning electronic microscopic pictures of flower details of *C. crepidioides* Ile-Ife. Side view of a closed capitulum **(G)**. Top view of a capitulum with different floret stages being visible **(H)**. Open anthers containing pollen **(I)**. The stigma of *C. crepidioides* with pollen attached **(J)**.

*Crassocephalum crepidioides* and *C. rubens* form composite flowers, which are comprised of disk florets that are united in capitula (also called pseudanthia) and are surrounded by involucral bracts (Sakpere et al., 2013). To investigate flower development in some detail, the capitula of two African accessions, *C*. *crepidioides* Ilé-Ifè (*C.c.*Ile-Ife) and *C. rubens* Mail (*C.r*.Mali), were analyzed at different stages by light and electron microscopy. This showed that flower development in both species began with the formation of flat buds, which elongated when the florets started to develop inside (stage 1–5; Figure 1A). In bud stage 6, the inner involucral bracts were almost fully developed and the florets started to elongate, with a strong expansion until stage 9 (Figure 1A and Supplementary Figure 1). When this stage was reached, the florets had fully developed and drops of liquid began to be exuded from the tips of the closed capitula (Figure 1B). This liquid was likely nectar secreted from the florets, a phenomenon also found in other plant species with composite flowers (Mesquita-Neto et al., 2020).

Anthesis took place at stage 10. The capitula opened at the top and the purple (*C. rubens*; Figures 1B–E) or brick–red (*C. crepidioides*; Figure 1F) tips of the florets showed. Each capitulum contained 120–160 disk florets, which were bisexual and opened from the outside to the inside (Figures 1F–H). The corolla was composed of five petals (Figure 1H) and was tubular, slender, and funnel-shaped. The tube had a white coloration at the base, which transcended to yellow in the upper half (Figure 1D) and ended in a purple (*C.r*.Mali) or red (*C.c.*Ile-Ife) limb. Stamens were fused into tubes (Figure 1I,J) with incised bases and purple (*C.r*.Mali) or red (*C.c.*Ile-Ife)-colored apexes. The stigma was bilobed (*C.r*.Mali) or monolobed (*C.c.*Ile-Ife) (Figures 1D–F) with penicillate tops (Figure 1J).

For fertilization, the mature florets released pollen into the anther column (Figure 1I), which was brushed by the stigma of the growing style to the distal opening (Figure 1J). Once all florets had opened and pollination had occurred, the inner phyllaries defused and the fruits developed.

**FIGURE 2:**
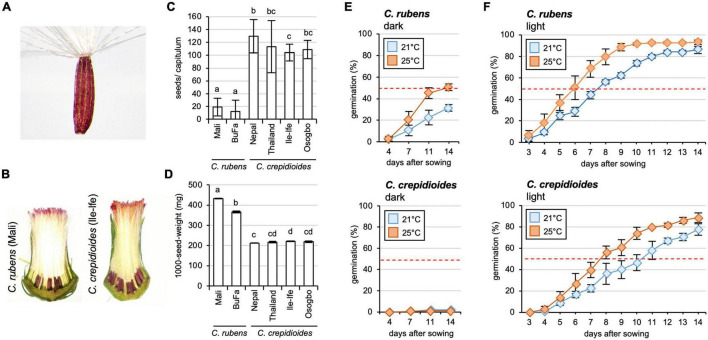
*Crassocephalum crepidioides* forms more seeds than *C. rubens*, which exhibit a higher level of seed dormancy. **(A)** Seed of *C. crepidioides* with hairy pappus. **(B)** Seed development in capitula of *C. rubens* and *C. crepidioides*. Sections of representative capitula are shown. **(C)** The number of seeds/capitulum of different accessions of *C. rubens* and *C. crepidioides*, grown in the greenhouse at 16 h light/8-h dark cycles. Twenty capitula were evaluated and the mean and SD are shown. Different letters over the error bars indicate significant differences (*p* < 0.05; ANOVA, *post hoc* Tukey). **(D)** One thousand-seed-weight of plants grown as in panel **(B)** (means ± SD; *n* = 4). Different letters over the error bars indicate significant differences (*p* < 0.05; ANOVA, *post hoc* Tukey). **(E,F)** Seed germination of *C. rubens* and *C. crepidioides* in the dark **(E)** or in the light **(F)**. Fifty seeds of *C. rubens* Mali (top) and *C. crepidioides* Ile-Ife (bottom) were plated on 1/2 MS and incubated in either the dark or in 16-h 30 μmol m^–2^s^–1^ white light/8-h dark cycles at a temperature of 21 or 25°C, respectively. Seed germination, defined as radicle emergence from the seeds coat, was evaluated at the indicated time points. Data show the mean ± SD with *n* = 50.

The fruits were cylindrical dark–brown ribbed achenes with a paler base and apex. They were thinly pubescent and attached to a pappus, which consisted of numerous translucent to white minutely toothed capillary bristles (Figure 2A). The time taken from bud formation to seed release differed between *C. rubens* and *C. crepidioides*, with approximately 7 weeks in the case of the former and approximately 6 weeks in the case of the later in greenhouse conditions (Figure 1A).

Interestingly, while similar numbers of florets were formed, much fewer seeds developed in the capitula of *C.r*.Mali than in those of *C.c.*Ile-Ife (Figure 2B). To quantify this observation and compare the fertility of *C. rubens* and *C. crepidioides* on a broader scale, additional ecotypes were included in the analyses, namely *C.c*.Osogbo, *C.c*.Nepal, *C.c*.Thailand, and *C.r*.Burkina Faso, and seeds per capitulum were counted. This showed that while *C. rubens* developed only about 20 seeds/capitulum, almost all florets were fertilized in *C. crepidioides*, yielding > 100 seeds/capitulum in the analyzed accessions (Figure 2C). The seeds formed by *C. crepidioides* were smaller and had a lower weight than those of *C. rubens* (Figures 2B,D).

### *Crassocephalum crepidioides* Seeds Exhibit a High Level of Seed Dormancy, Which Is Conferred by Abscisic Acid

*Crassocephalum crepidioides* had previously been shown to have a light requirement for germination (Chen et al., 2009; Nakamura and Hossain, 2009; Sakpere et al., 2013; Yuan and Wen, 2018). To verify this, germination experiments with seeds of *C.r*.Mali and *C.c.*Ile-Ife with the same growth history were carried out. Fifty seeds of each accession were sterilized, plated on 1/2 MS medium, incubated in the dark or 16 h of white light/8-h dark at two different temperatures (21 or 25°C), and germination was evaluated at different time points. The results showed that in the dark, seeds of both species exhibited a high level of dormancy. Whereas *C. crepidioides* seeds had no germination capacity in the dark, *C. rubens* seeds germinated with approximately 30% efficiency (Figure 2E). Light promoted germination, with stronger effects on *C. rubens* seeds, which reached 50% germination already after 7 days, whereas *C. crepidioides* seeds took 10 days at 21°C (Figure 2F). Increasing the temperature to 25°C clearly promoted germination in both species, yielding close to 100% germination after 10 days in the case of *C. rubens* and after 14 days in the case of *C. crepidioides* (Figure 2F).

Plant hormones play an important role in seed germination, with gibberellins (GAs) and brassinosteroids (BRs) promoting and ABA impairing it in *Arabidopsis thaliana* and other plant species (Bentsink and Koornneef, 2008; Unterholzner et al., 2015). To investigate if these hormones may also play a role in the germination of *Crassocephalum* seeds, the BR 24-epi Brassinolide (epiBL), the GA GA_3_, and ABA were added to the 1/2 MS medium in different concentrations and germination outcomes were analyzed. In addition, also inhibitors of hormone activity were applied, namely, Brassinazole (BRZ; Asami et al., 2000) an inhibitor of BR biosynthesis, and Fluridone (Flu; Bartels and Watson, 1978; Kondhare et al., 2014), an inhibitor of ABA biosynthesis. The results showed that whereas epiBL and GA_3_ had no significant effect (Supplementary Figures 2A,B), the BR biosynthesis inhibitor BRZ inhibited germination in both species (Supplementary Figure 2C) indicating that BRs promote the germination of *Crassocephalum* seeds.

**FIGURE 3:**
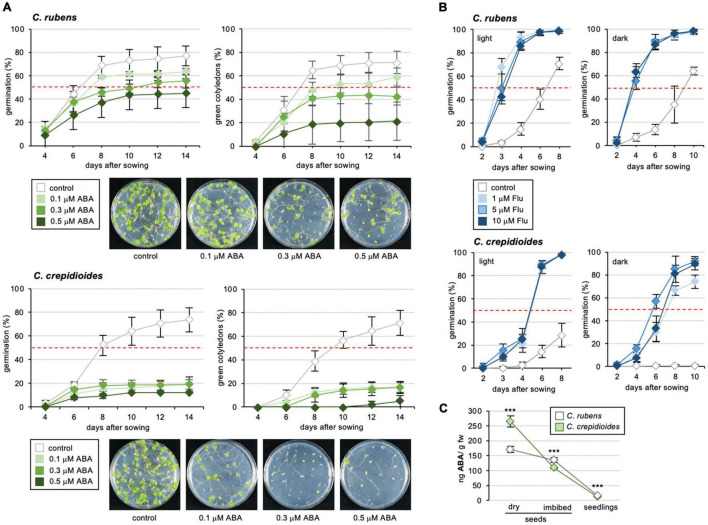
*Crassocephalum crepidioides* is hypersensitive to ABA and Fluridone treatment during germination. **(A)** Response of *C. rubens* and *C. crepidioides* to externally applied ABA in terms of germination capacities and cotyledon greening. 50 seeds of each accession were plated on 1/2 MS medium without (control) or with 0. 1-, 0. 3-, or 0.5-μM ABA and incubated in 16-h 30 μmol m^–2^s^–1^ white light/8-h dark cycles at a temperature of 25°C. Seed germination (left graph), was defined as radicle emergence from the seeds coat. Cotyledon greening (right graph) was defined as fully developed, green cotyledons. The means and SDs are shown. Photos of representative plates are shown below the graphs. **(B)** Response of *C. rubens* and *C. crepidioides* seeds to externally applied Flu during germination in the light and in the dark. 50 seeds of each accession were plated on 1/2 MS medium without (control) or with 1-μM Flu, 5-μM Flu, or 10-μM Flu and incubated in 16-h 30 μmol m^–2^s^–1^ white light/8-h dark cycles (left graph) or in the dark (right graph) at a temperature of 25°C. **(C)** The ABA levels in seeds and seedlings of *C. crepidioides* and *C. rubens*. The ABA levels were determined using LC/MS-MS in fresh dry seeds, fresh seeds imbibed for 24 h in water and 3-day-old seedlings; *n* = 4–5. In all charts, the means and SDs are shown. In all graphs, the asterisks indicate significant differences compared to the wild-type by Student’s *t*-test (***p ≤ 0.001).

Abscisic acid impaired germination in both *C.r.*Mali and *C.c.*Ile-Ife, but with much stronger effects on the latter (Figure 3A), for which already a concentration of 0.1 μM ABA reduced germination in the light to 20% at 14 days post-incubation. To analyze this ABA hypersensitivity further, cotyledon greening was assessed, an assay routinely used to quantify ABA responsiveness (Lopez-Molina et al., 2001). This showed that seedlings of *C. crepidioides* were extremely sensitive to ABA, with 0.1 μM ABA reducing greening of *C.c.*Ile-Ife to below 20%, an amount were cotyledons of *C.r.*Mali had a greening rate of 60% after 14 days of incubation (Figure 3A).

These results suggested that in *C. crepidioides*, ABA biosynthesis and/or responses are increased, which may explain aspects of the low germination capacities of *C. crepidioides* seeds, especially in the dark. To address, if this may relate to increased ABA biosynthesis, germination outcomes were evaluated in the presence of Flu, which showed that Flu clearly improved germination of both *C. rubens* and *C. crepidioides* (Figure 3B). The effects were particularly striking in the case of *C. crepidioides*, where Flu completely restored germination in the dark, indicating that increased ABA levels may account for the high degree of seed dormancy in this species.

To verify this, levels of ABA were measured by liquid chromatography tandem mass spectrometry (LC-MS/MS) in dry seeds, imbibed seeds, and 3-day-old seedlings, which showed that concentrations of ABA were significantly higher in dry seeds of *C. crepidioides* than in *C. rubens*, but dropped to similar levels after imbibition (Figure 3C). Therefore, in summary, seeds of *C. crepidioides* exhibit a higher level of seed dormancy than those of *C. rubens*, which is caused by elevated levels of ABA and an ABA hyper-responsiveness and can be broken with the ABA biosynthesis inhibitor Flu, and with light.

### *Crassocephalum crepidioides* Exhibits a Higher Level of Drought and Heat Stress Tolerance Than *Crassocephalum rubens*

**FIGURE 4:**
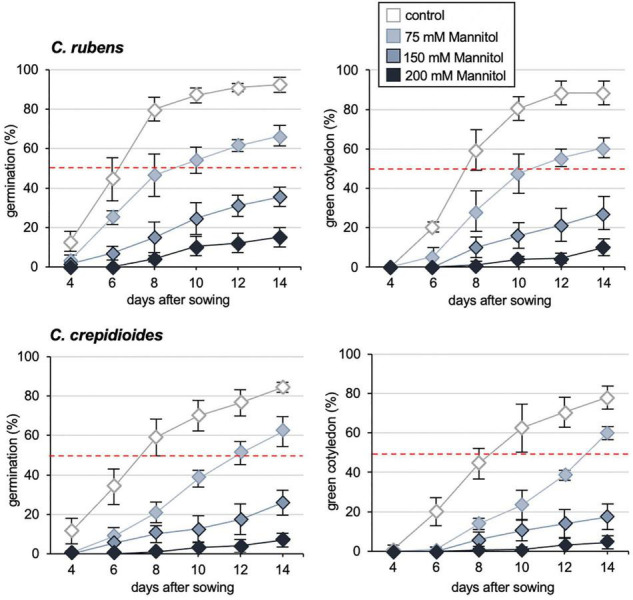
*Crassocephalum crepidioides* shows increased resistance to Mannitol. Response of *C. rubens* Mali and *C. crepidioides* Ile-Ife to externally applied Mannitol in terms of germination capacities and cotyledon greening. Fifty seeds of each accession were plated on 1/2 MS medium without (control) or with 0, 75, 150, and 200 mM of Mannitol and incubated in 16-h 30 μmol m^–2^s^–1^ white light/8-h dark cycles at a temperature of 25°C. Seed germination (left graphs) was defined as radicle emergence from the seeds coat. Cotyledon greening (right graphs) was defined as fully developed, green cotyledons. The means and SDs are shown.

In addition to dormancy, ABA plays a major role in abiotic stress responses of plants (Nakashima and Yamaguchi-Shinozaki, 2013) and thus we asked, if the increased ABA responses of *C. crepidioides* may benefit abiotic stress tolerance of this species. To investigate this, germination and cotyledon greening assays were carried out in the presence of sodium chloride (NaCl) or Mannitol, which are compounds that induce ABA-conferred signaling responses for protection from osmotic stress (Fujita et al., 2011; Yoshida et al., 2014). The results showed that whereas NaCl responses were similar (Supplementary Figure 3), *C. crepidioides* was hyper-responsive to Mannitol as compared to *C. rubens*, with particularly clear differences at a concentration of 75 mM (Figure 4), indicating altered tolerance to stress types that induce cellular desiccation.

**FIGURE 5:**
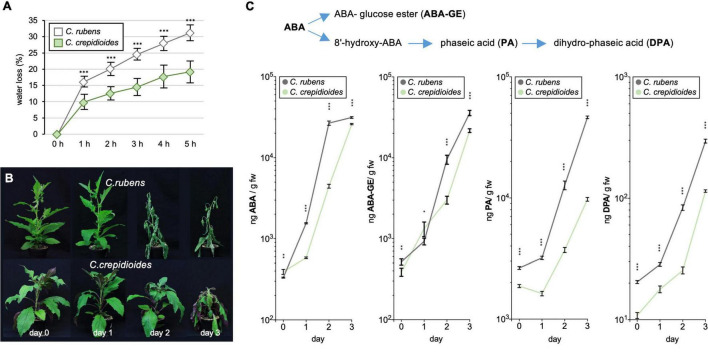
*Crassocephalum crepidioides* shows reduced leaf water loss and delayed accumulation of ABA and its catabolites as compared to *C. rubens*. **(A)** Detached leaf water loss assays with leaves of 4-week-old *C. rubens* Mali and 5-week-old *C. crepidioides* Ile-Ife plants, grown in 16 h 80 μmol m^–2^s^–1^ white light/8-h dark cycles at a temperature of 25°C; *n* = 10. **(B,C)** The ABA measurements in drought-stressed plants. **(B)** Seven-week-old *C. rubens* Mali and 8-week-old *C. crepidioides* Ile-Ife plants, grown as in A, were subjected to progressive drought by withholding water for 3 days. Photos of representative plants are shown. **(C)** Levels of ABA, ABA-GE, PA, and DPA were then measured in these plants with LC-MS/MS; *n* = 4–5. In all graphs, the data show the mean ± SD; the asterisks indicate significant differences between genotypes by Student’s *t*-test (*p ≤ 0.05; ^**^p ≤ 0.01; ^***^p ≤ 0.001).

To investigate this further, detached leaf water loss assays were conducted, to evaluate water loss over time, a parameter that contributes to drought tolerance. The results showed that *C. rubens* lost water significantly faster than *C. crepidioides* (Figure 5A), indicating decreased stomatal guard cell conductivity in response to drought. Stomatal conductance is controlled by ABA (Hsu et al., 2021) and to test, if increased ABA concentrations may account for the reduced water loss in *C. crepidioides*, levels of ABA and its catabolites phaseic acid (PA), dihydrophaseic acid (DPA), and ABA glucose ester (ABA-GE) were measured daily in adult plants of *C. rubens* and *C. crepidioides* after water withdrawal for 3 days (Figure 5B). Whereas *C. crepidioides* exhibited higher ABA levels at day 0, the faster water loss of *C. rubens* was correlated with a more rapid increase in ABA and its catabolites (Figure 5C), providing evidence that it is not a decreased ability to synthesize ABA, which accounts for the increased water loss of *C. rubens*.

**FIGURE 6:**
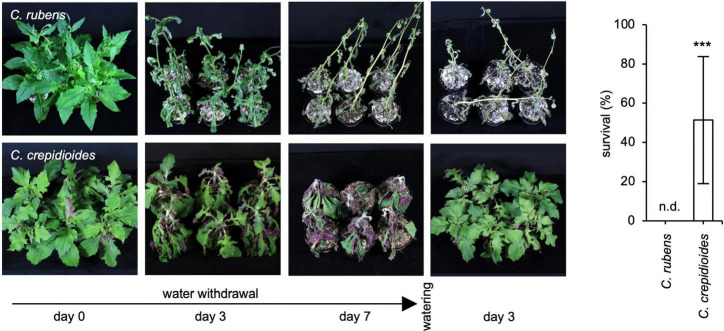
*Crassocephalum crepidioides* shows a higher recovery potential following drought than *C. rubens.* Seven-week-old plants of *C. rubens* Mali and 8-week-old plants of *C. crepidioides* Ile-Ife, grown in 16-h white light/8-h dark cycles at 25°C, were subjected to progressive drought by withholding water for 7 days before they were re-watered. Left: Photos of representative plants, taken at the indicated time-points. Right: Survival rates, which are defined as the ability to form new leaves. The mean and SD of three biological replicates, with at least 12 individual plants each, are shown. The asterisk indicates significant differences between genotypes by Student’s *t*-test (***p ≤ 0.001); n.d.: not detected.

The ABA measurements showed that the effects of water withdrawal on ABA formation were delayed in *C. crepidioides*. To assess, if this impacts the drought stress tolerance of the species, a drought stress experiment was carried out, in which water was withheld for 7 days and then the plants were re-watered. Importantly, whereas *C. rubens* had died off, *C*. *crepidioides* recovered (Figure 6), further supporting the idea that it has an elevated drought stress tolerance.

Since ABA can also impact the heat and cold stress resistance of plants (Nakashima and Yamaguchi-Shinozaki, 2013; Huang et al., 2016; Albertos et al., 2022), we compared the ability of *C. rubens* and *C. crepidioides* to cope with temperature extremes. While both species survived freezing temperatures of –4°C for 3 h to a similar extent (Supplementary Figure 4), *C*. *crepidioides* exhibited a higher heat stress tolerance, surviving 5 h of 45°C, a treatment that *C*. *rubens* did not survive (Supplementary Figure 5).

## Discussion

*Crassocephalum crepidioides* and *C. rubens* are orphan crops with high potential to improve crop diversity and nutrition in countries of Sub-Saharan Africa. The plants are still mainly collected from the wild but shall be taken into cultivation and an important aim in the domestication of these species is to improve traits that are relevant for crop production and food safety. In the context of food safety, it has previously been shown that *C. crepidioides* can form the highly toxic PA jacobine, an ability that *C. rubens* lacked, at least under the tested conditions (Schramm et al., 2021). Given the presence of this anti-nutritional trait, it is relevant to ask, if *C. crepidioides* is a sensible choice for food production, or if it should rather be *C. rubens*, our activities should be focused on. Therefore, in this work, the two species were compared, to obtain more information about potential differences in relevant traits, such as seed germination, yield quantity, and yield stability in presence of abiotic stress.

*Crassocephalum crepidioides* is tetraploid and polyploids can have competitive advantages over their diploid relatives (Comai, 2005). Such advantages include the ability to form a richer spectrum of secondary metabolites and in line, *C. crepidioides* formed more jacobine than *C. rubens*, but also accumulated more anthocyanins in stressful conditions, such as under nitrogen starvation and when grafted (Schramm et al., 2021). In addition, polyploids can show higher biomass gains and seed yields (Comai, 2005; Mayfield et al., 2011; Wei et al., 2019), traits that are beneficial for plant production, and previously it was shown that *C. crepidioides* has delayed flowering and thus a prolonged vegetative growth phase, resulting in larger biomass gains as compared to *C. rubens* (Schramm et al., 2021). Here it was found that also seed yield was clearly increased in *C. crepidioides*, which formed approximately 100 seeds per capitulum, whereas diploid *C. rubens* formed only about 20 seeds. This was due to impaired seed development since in the capitula of *C. rubens* many ovules arrested and did not develop into seeds. At the moment it is not clear, whether this is caused by a reduced rate of pollination, fertilization, or less efficient embryo or endosperm development. While fewer seeds were formed, the weight of the individual seeds was higher in the case of *C. rubens*, with approximately 0.40 g (0.36 to 0.43 g and for the two accessions), whereas it was only approximately 0.22 g for all *C. crepidioides* accessions, the latter being in line with previous results (Denton, 2004; Chen et al., 2009; Adjatin et al., 2013; Sakpere et al., 2013).

Seed formation in *C. rubens* also took longer than in *C. crepidioides* in the tested conditions, and this was observed in all the accessions. In *C. crepidioides*, seeds were fully developed approximately 9 days after the opening of the capitula, whereas it took 16 days in *C. rubens*. In line, also the total time it took from capitula formation to seed release differed between *C. rubens* and *C. crepidioides. C. crepidioides* took approximately 40 days, whereas *C. rubens* took approximately 50 days in the applied conditions.

In addition to seed quantity, another important trait that requires attention is seed quality and in *Crassocephalum* this relates to seed viability and germination capacities since they can limit the cultivation of *C*. *crepidioides* (Sakpere et al., 2013). The plant is a pioneer species that rapidly colonize open areas through wind-dispersal of its seeds and there was the first evidence that *C. crepidioides*, like other pioneer plants, has a light-requirement for germination (Chen et al., 2009; Nakamura and Hossain, 2009; Sakpere et al., 2013; Yuan and Wen, 2018). To address this, the Nigerian *C. crepidioides* ecotype *C.c*.Ile-Ife and the *C. rubens* ecotype *C.r*.Mali were compared, which confirmed that on 1/2 MS medium, *C. crepidioides* was unable to germinate in the dark, whereas *C. rubens* showed some germination capacities, albeit to a very low extend.

Light very efficiently promoted germination of *C. crepidioides* and *C. rubens* seeds, with the effects being stronger in the case of *C. rubens*, which reached about 50% germination already after 7 days, whereas *C. crepidioides* took 10 days at 21°C. Increasing the temperature to 25°C also promoted germination in both species, with clearer effects for *C. rubens*, which achieved close to 100% germination already after 10 days, whereas *C. crepidioides* took 14 days.

It is very well established that the germination outcomes in plants are highly controlled by diverse environmental cues, with light playing a key role. Light is perceived by photoreceptors that specifically detect certain wavelengths, and for red/far–red light perception the phytochromes are utilized, which release photo-dormancy in most light-requiring seeds. Phytochromes perceive light to activate hormone biosynthetic and signaling pathways, which convey physiological responses and during seed germination in particular the role of GA is very well established (Neff et al., 2000; Weitbrecht et al., 2011). Phytochromes activate seed germination through the induction of GA metabolism and responses, and in line with GAs acting downstream of light perception, GA application promotes germination in many plants, even if the light environment is not ideal. However, in *Crassocephalum*, the application of the GA GA_3_ had no impact on seed germination, even at a relatively high concentration of 10 μM, which may indicate that GAs do not play a major role. Alternatively, and more likely, the GA_3_ concentration may have been too low, GA_3_ may not have been taken up, or may not be able to efficiently activate GA responses in these species. Also, the BR 24-epiBL, which can promote germination in other species (Tong et al., 2014; Unterholzner et al., 2015), showed no effect on *C. rubens* or *C. crepidioides* germination capacities, which may have similar reasons.

While GA_3_ and 24-epiBL did not have a significant impact, ABA suppressed seed germination in both *C. crepidioides* and *C. rubens*, with stronger effects on *C. crepidioides*. In addition, C. *crepidioides* was also hyper-responsive to ABA in terms of its ability to suppress cotyledon greening. Moreover, an application of the ABA biosynthesis inhibitor Flu markedly improved the germination of both *C. rubens* and *C. crepidioides.* This activity was particularly impressive in *C. crepidioides*, where the lack of germination capacities in the dark was completely restored with Flu. Conclusive evidence for an altered ABA biosynthesis came from LC-MS/MS measurements of ABA in dry seeds, imbibed seeds, and 3-day-old seedlings. Abscisic acid was higher in dry seeds of *C. crepidioides* than in those of *C. rubens* but dropped to similar levels after imbibition.

In other plant species, it is well established that seed dormancy is released upon imbibition through ABA degradation (Gubler et al., 2005) and this was also correlated well in *Crassocephalum*. After imbibition, the amount of ABA accumulated in dry seed rapidly declines, and this is achieved through catabolism of ABA to form hydroxylated or glucosylated ABA (Sano and Marion-Poll, 2021). ABA-GE levels quickly dropped during imbibition in both *C. crepidioides* and *C. rubens*. Thus, there is clear evidence that *C*. *crepidioides* has an increased seed dormancy as compared to *C. rubens*, which correlates with increased ABA levels and hyper-responsiveness to ABA. Light and Flu can break seed dormancy in *Crassocephalum*, which is a relevant finding for plant production.

Since ABA plays a vital role in abiotic stress responses of plants, it was investigated if the increased ABA responses of *C. crepidioides* may benefit the abiotic stress tolerance of this species. Germination and cotyledon greening experiments in the presence of NaCl or Mannitol showed that while NaCl responses were comparable, *C. crepidioides* was hyper-responsive to Mannitol. Thus, there was evidence that resistance against stress types that induce cellular desiccation is increased. To study this further, detached-leaf water-loss assays were conducted and showed that *C. rubens* lost water faster and also wilted earlier than *C. crepidioides*, indicating that the latter has an increased stomatal guard cell conductivity.

As the stomatal conductance is controlled by ABA (Hsu et al., 2021), ABA was measured in drought-stressed plants, which showed that the reduced water loss in *C. crepidioides* correlated with higher ABA levels before the onset of stress but did not result in enhanced ABA formation, but rather with a less rapid increase in the ABA contents in the leaves. In addition, also the formation of the analyzed ABA catabolites was delayed in *C. crepidioides*, indicating that the stress response kicked in later, potentially due to the fact that the plants were less damaged by drought. To verify this, it was tested if the recovery potential after 7 days of drought exposure differed between the two species, by re-watering the plants. This revealed that whereas *C*. *rubens* was unable to recover and died off, *C. crepidioides* showed an impressive ability to survive this period of water withdrawal. Therefore, even though *C. crepidioides* produces more biomass, it can survive with less amount of water than *C. rubens*, at least for a limited period of time, another very relevant trait for plant production.

In addition to increasing drought tolerance, ABA can promote cold stress tolerance (Eremina et al., 2016), but appears to repress resistance to heat stress (Larkindale et al., 2005; Huang et al., 2016; Albertos et al., 2022), and it was therefore interesting to investigate, how well *Crassocephalum* species can cope with temperature extremes. Both species survived freezing temperatures of –4°C for 2 h, which is surprising given that they originate from subtropical zones. *Crassocephalum crepidioides*, however, displayed a higher heat stress resistance, surviving 5 h of 45°C, a temperature that *C. rubens* could not survive.

In conclusion, this and previous work showed that *C. crepidioides* outperformed *C. rubens* in a number of traits, including biomass gains, seed yield, drought tolerance, and heat stress tolerance, which may relate to its tetraploid nature. A high degree of seed dormancy and drought tolerance was correlated with an ABA hyper-sensitivity and this may contribute to the higher survival potential of *C. crepidioides* in stress-full conditions. In particular, the high drought tolerance and heat stress tolerance will benefit *C. crepidioides* production in rain-fed agriculture. Thus, although it accumulates PAs, this tetraploid species warrants further work, to exploit its potential for combating malnutrition and increasing the resilience of marginal cropping systems in Africa.

## Data Availability Statement

The raw data supporting the conclusions of this article will be made available by the authors, without undue reservation.

## Author Contributions

AA-B and BP planned and designed the research. AA-B, SS, MG, and WI contributed to the performance of research. BP wrote the manuscript with major contributions from AA-B. All authors analyzed the data and contributed equally to finalize the manuscript.

## Conflict of Interest

The authors declare that the research was conducted in the absence of any commercial or financial relationships that could be construed as a potential conflict of interest.

## Publisher’s Note

All claims expressed in this article are solely those of the authors and do not necessarily represent those of their affiliated organizations, or those of the publisher, the editors and the reviewers. Any product that may be evaluated in this article, or claim that may be made by its manufacturer, is not guaranteed or endorsed by the publisher.
