# Supplementary material for: Species-Specific Variation in Abscisic Acid Homeostasis and Responses Impacts Important Traits in Crassocephalum Orphan Crops
Source: Front Plant Sci. 2022 Jul 12;13:923421. doi: 10.3389/fpls.2022.923421 (PMC9318166; doi:10.3389/fpls.2022.923421)
Supplement: Supplementary file 1 [file Data_Sheet_1.PDF]

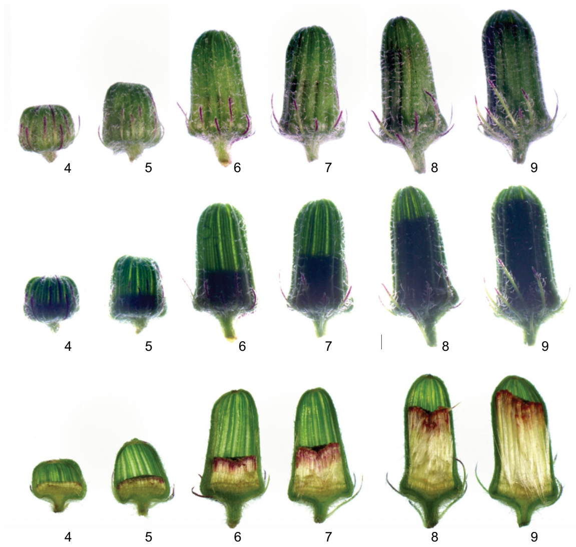

SUPPLEMENTARY FIGURE 1. Floret development in capitula of *C. crepidioides* Ile-Ife stages 4-9.

In the top and middle row photos of capitula are shown, that were illuminated either from the top or from the bottom. In the bottom row sections are shown.

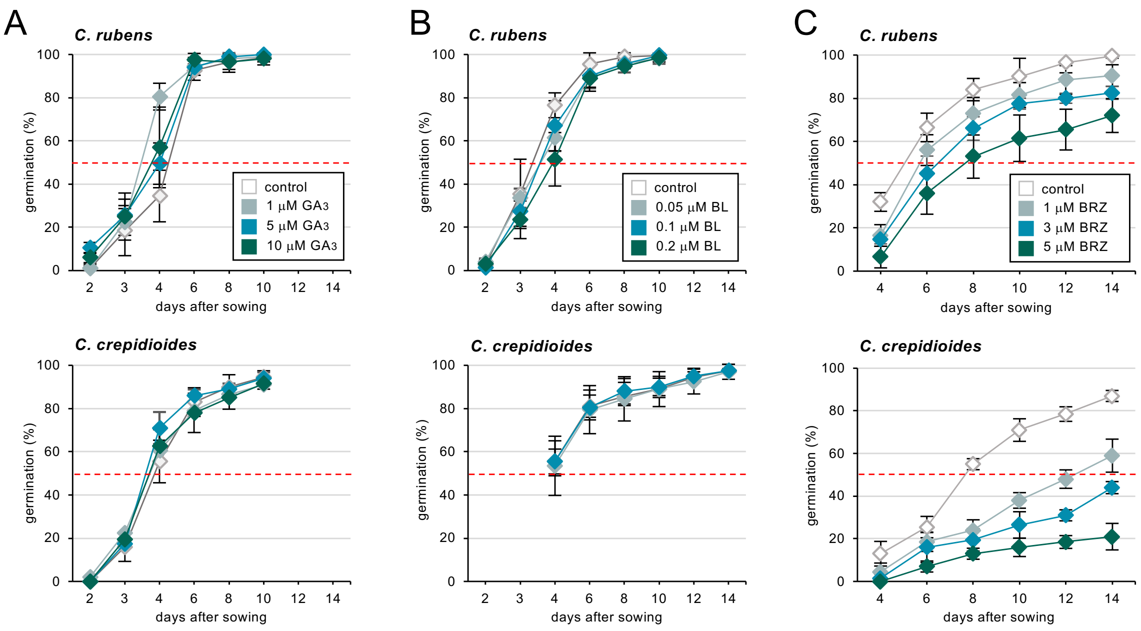

SUPPLEMENTARY FIGURE 2. Seed germination of *C. rubens* and *C. crepidioides* in presence of GA_3, 24-epiBL or BRZ.

50 seeds of *C. rubens* Mali and *C. crepidioides* Ile-Ife were plated on ½ MS medium without (control) or with different concentrations of GA_3 (**A**), 24epiBL (**B**) or BRZ (**C**) and incubated in 16 hour 30 µmol/m^2s^1 white light/ 8 hour dark cycles at a temperature of 25˚C. Seed germination, defined as radicle emergence from the seeds coat, was determined at the indicated time points. Data show the mean ±SD.

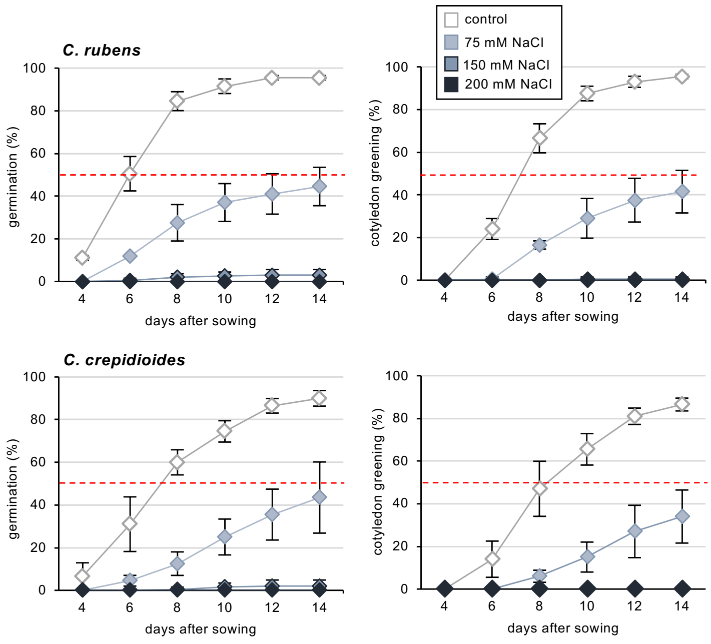

SUPPLEMENTARY FIGURE 3. Seed germination of *C. rubens* and *C. crepidioides* in presence of NaCl.

50 seeds of *C. rubens* Mali and *C. crepidioides* Ile-Ife were plated on ½ MS medium without (control) or with 75, 150 and 200 mM NaCl and incubated in 16 hour white light (30 µmol/m^{2}s^1)/ 8 hour dark cycles at a temperature of 25˚C. Seed germination, defined as radicle emergence from the seeds coat (left), and cotyledon greening, defined as fully developed green cotyledons (right) were determined at the indicated time points. Data show the mean ±SD.

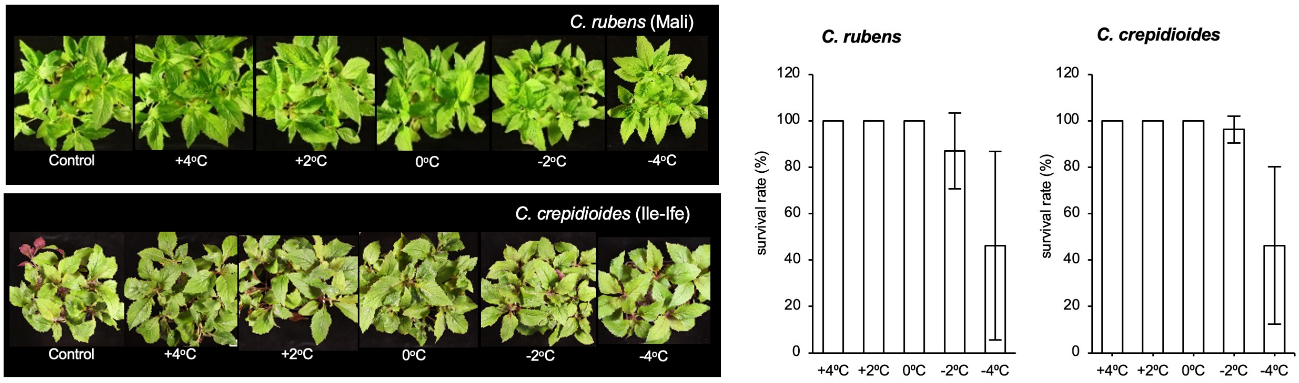

SUPPLEMENTARY FIGURE 4. Frost tolerance of *C. rubens* and *C. crepidioides.*

Four-week-old plants of *C. rubens* Mali and *C. crepidioides* Ile-Ife, grown in 16 hour 80 µmol/m^2s^1 white light/ 8 hour dark cycles at a temperature of 25˚C, were exposed to the indicated temperatures for 3 hrs. Left: Photos of representative plants. Right: Quantification of survival rates, as defined by the ability to form intact SAMs. Data show the mean ±SD with n=5.

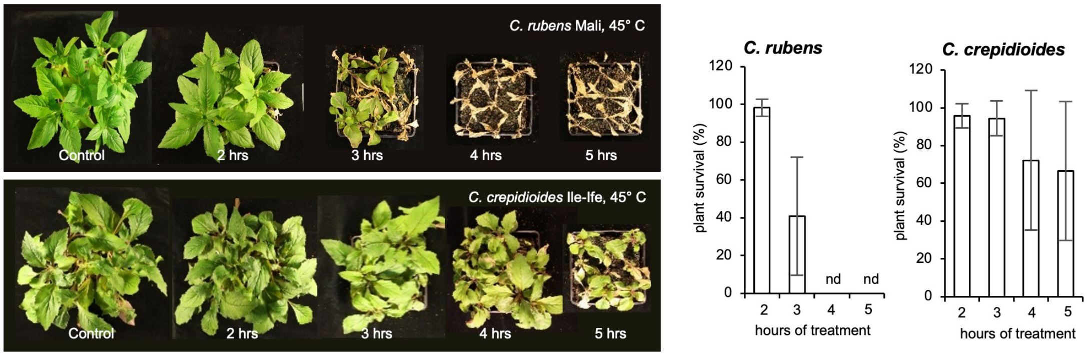

SUPPLEMENTARY FIGURE 5. *C. crepidioides* is more resistant to heat stress than *C. rubens*.

Four-week-old plants of *C. rubens* Mali and *C. crepidioides* Ile-Ife, grown in 16 hour 80 μmol/m^{2}s^1 white light/ 8 hour dark cycles at a temperature of 25˚C, were exposed to 45 °C for the indicated periods of time. Left: Photos of representative plants. Right: Quantification of survival rates, as defined by the ability to form intact SAMs. Data show the mean ±SD with n=5.
